# Exploration of the role of Cuproptosis genes and their related long non-coding RNA in clear cell renal cell carcinoma: a comprehensive bioinformatics study

**DOI:** 10.1186/s12885-022-10278-z

**Published:** 2022-11-06

**Authors:** Dian Xia, Qi Liu, Wen Jiao, Longfei Peng, Qi Wang, ZhouTing Tuo, Liangkuan Bi

**Affiliations:** 1 Department of Urology, The Second Hospital of Anhui Medical University, Hefei, China; 2 Department of Oncology, The First Affiliated Hospital of Anhui Medical University, Hefei, China; 3 Department of Urology, Peking University Shenzhen Hospital, ShenZhen, China

**Keywords:** Renal clear cell carcinoma, Cuproptosis, Long non-coding RNA, Prognosis, Immunity therapy, Bioinformatics engineering

## Abstract

Clear cell renal cell carcinoma is a common malignant tumor of the urinary system. The mechanism of its occurrence and development is unknown, and there is currently few effective comprehensive predictive markers for prognosis and treatment response. With the discovery of a new cell death process – cuproptosis drew the attention of researchers. We constructed a model for the prediction of clinical prognosis and immunotherapy response through integrative analysis of gene expression datasets from KIRC samples in The Cancer Genome Atlas (TCGA) database. During the course of the study, we found that cuproptosis genes are significantly differentially expressed between clear cell renal cell carcinoma samples and normal samples. Based on this, we put forward the prognostic model for cuproptosis gene related-long non-coding RNA. And through various statistic and external independent cohorts, we proved that the model is accurate and stable, worthy of clinical application and further exploration and validation.

**Supplementary Information:**

The online version contains supplementary material available at 10.1186/s12885-022-10278-z.

## Introduction

Renal cell carcinoma (RCC) is the most common malignancy in the kidneys. There are about 210,000 new patients with this disease worldwide each year, accounting for 2–3% of all cancer cases. Kidney renal clear cell carcinoma (KIRC) or clear cell renal cell carcinoma (ccRCC) is a main histological subtype of RCC, accounting for 80–90% of the total number of RCC patients. There is a poor prognosis for patients with KIRC, which seriously affects their life and health [1]. Although surgical treatment is effective in the treatment of patients with kidney cancer at an early stage, the recurrence and metastasis may occur in as many as 30% of patients after radical surgery, who have unfavorable survival and prognosis [2]. Generally, the patient with metastatic renal cell carcinoma (Mrcc) cannot be cured, with the median survival being only 18 months and a low 5-year survival rate. In recent years, some patients with kidney cancer have benefited from the immune checkpoint inhibitors, especially the programmed death receptor-1 (PD-1) and its ligand (PD-L1) inhibitors [3]. However, the overall effective rate of immunotherapies is less than 40%, and a considerable number of patients cannot benefit from immunotherapies [4]. As per some analysis results, in addition to the low sensitivity of patients with kidney cancers to immunosuppressants, drug resistance in tumors is also a common reason for the decreased treatment efficiency. Therefore, the survival and prognosis of patients with kidney cancer can be effectively improved by exploring the important biological processes in the occurrence and development of kidney cancer and identifying drugs sensitive to tumor treatment.

The long non-coding RNA (lncRNA) is an RNA with a length of more than 200 bp that cannot encode proteins, and it is extensively distributed in the nucleus and cytoplasm [5]. In previous, lncRNAs were thought to be the “noise” in the process of gene expression [6]. However, DERRIEN et al. [7] found that lncRNAs are produced through a transcriptional pathway similar to that of the coding gene and have similar histone modifications, splicing patterns and exons/ introns. LncRNAs are transcribed from either strand of the coding gene, and they can or not be polyadenylated [8]. Currently, it has been confirmed in related studies that lncRNAs have a decisive role in RCC. WANG et al. [9] found that the lncRNA RP11-436H11.5 can be overexpressed in kidney cancer cells OSRC-2, the expression level of the oncogene BCL-W protein is elevated and cell invasion is also enhanced. After these cells are treated with the BCL-W inhibitor ATB-737, cell invasion is reduced; the inhibition is more pronounced at a higher concentration of ATB-737. Meanwhile, HE et al. [10] analyzed the tissue and plasma samples from 46 patients with RCC, and they found that lncRNA GIHCG increases significantly in the tissue and plasma samples of these patients (*P* < 0.01). The lncRNA GIHCG in stage II-IV is significantly higher than that in stage I (*P* = 0.028). Besides, the lncRNA GIHCG in Fuhrman G3-G4 is significantly higher than that in Fuhrman G1-G2 (*P* = 0.032).

All life activities can be traced back to cell metabolism, which provides an energy source and material basis for cell growth and proliferation. Multiple complex metabolic enzymes may generate abundant small molecules of metabolites during cell metabolism. These small molecules not only exert influence in the classical metabolic pathway, but also fulfill a non-metabolic function as signal molecules. These molecules could connect the extracellular microenvironment factors with the intracellular gene expression information, which would exercise an impact on various features and processes of cells, thus affecting the occurrence and development of tumors.

Copper is an indispensable molecule in cell metabolism. According to recent studies, cuproptosis is induced by its direct combination with components related to lipidation in the tricarboxylic acid cycle (TCA cycle). It may cause the aggregation of 3apidated proteins and the loss of iron-sulfur cluster proteins, which would induce protein toxic stress and cell death in the end [11]. As is known to all, the mitochondrion is the energy metabolism center of cells, and the TCA cycle in mitochondria is a common metabolic pathway in aerobic organisms [12, 13]. According to the findings of this study, it can be speculated that cuproptosis, which is different from pyroptosis and ferroptosis, may operate a larger function in predicting the survival of tumor cells and the occurrence and development of tumors. It has been revealed from previous studies that copper ions can be enriched significantly in tumor cells compared with normal cells, but the content of other metal ions (such as iron and zinc ions) is usually lower than the normal value [14]. In addition, copper can also promote the occurrence and development of tumors by enhancing the metastasis of cancer cells and activating cell proliferation and metabolism [15]. Supported by the above evidences, we took the lead in using bioinformatics technology to explore the expression differences of cuproptosis-related genes in KIRC tissues, and constructed a clinical prediction model for cuproptosis gene-related long non-coding RNA. In addition, we conducted further exploratory analyses of tumor immune response and mutation load based on this model.

## Materials and methods

### Raw data

The transcriptome RNA-seq data, corresponding clinical data and mutation data of KIRC cases were downloaded from the TCGA database (https://portal.gdc.cancer.gov/) with API v3.0.0. (Data Release 31.0 – October 29, 2021). The validation cohort is the expression profile data of renal cell carcinoma in International Cancer Genome Consortium(ICGC) database(https://dcc.icgc.org/), a total of 91 samples of renal cell carcinoma.

### Identification of cuproptosis-related prognostic lncRNAs

A total of 19 cuproptosis-related genes were retrieved from previous research and literature; they are shown in Supplementary Table S1. We screened the lncRNAs in the TCGA cohort according to gene annotation and obtained a total of 2876 lncRNAs. The Pearson correlation coefficient was conducted to evaluate the correlation between 19 cuproptosis-related genes and lncRNAs. The lncRNA with an absolute correlation coefficient > 0.4 and a *P* value < 0.001 was considered as a cuproptosis-related lncRNA, and we screened a total of 197 cuproptosis-related lncRNAs. Then, a univariate Cox regression analysis of OS was performed to screen cuproptosis-related lncRNAs with prognostic value; *P* < 0.05 was considered.

to be related to the prognosis, a total of 111 cuproptosis-related lncRNAs with prognostic value were screened.

### Constructing and evaluating prognostic model

The model was constructed in the light of the training set; meanwhile, the testing set and the entire set were applied to test the predicted ability of the model. Multivariate Cox regression analysis was conducted in the training set and identified 8 cuproptosis-related lncRNAs, We named this model as “CLRM.” The following formula was employed to evaluate the risk score: risk score = coef (lncRNA1)*expr (lncRNA1) + coef (lncRNA2) * expr (lncRNA2) + …… + coef (lncRNAn)* expr (lncRNAn), where coef means the coefficients, coef (lncRNAn) indicates the coefficient of lncRNAs related to survival, and expr (lncRNAn) is the expression of lncRNAs.

### Exploration of the model in the immunotherapeutic treatment

The mutation data were evaluated and calculated with the assistance of the R package maftools. The tumor mutation burden(TMB) was measured according to tumor-specific mutated genes. Further, the Tumor Immune Dysfunction and Exclusion (TIDE) algorithm was adopted to predict the likelihood of the immunotherapeutic response [16].

### PCA and Kaplan–Meier survival analysis

Principal component analysis(PCA) was conducted on effective dimensionality reduction, model identification, and grouping visualization of high-dimensional data of the entire gene expression profiles, cuproptosis-related genes, cuproptosis-related lncRNAs, and risk model according to the expression patterns of the 8 cuproptosis-related lncRNAs [17]. Additionally, Kaplan–Meier survival analysis was also conducted to appraise diversities in the OS between both groups. The R packages survMiner and survival were adopted in this process.

### Exploration of potential compounds targeting the Cuproptosis-related lncRNAs Risk Model (CLRM) in clinical treatment

In an attempt to identify potential compounds in clinical treatment of KIRC patients, the IC50 of compounds obtained from the Genomics of Drug Sensitivity in Cancer (GDSC) website in the TCGA project of the KIRC dataset was calculated. The R package pRRophetic was used to predict the IC50 of compounds obtained from the GDSC website in patients with KIRC.

### Independence of the CLRM

Multivariate and univariate Cox regression analyses were conducted to test whether the prognostic pattern was a variable independent of other clinical features (age, gender, stage and grade) in the patients with KIRC [18].

### Establishment and verification of a predictive nomogram

The predictive ability of the nomogram and other predictors (age, gender, stage, grade and risk score) for the 1-, 3-, and 5-year OS was established. The correction curves based on the Hosmer–Lemeshow test were adopted to illustrate the uniformity between the practical outcome and model prediction outcome.

### Comprehensive analysis of molecular and immune characteristics and Immune checkpoint inhibitors(ICIs) therapy in different CLRM risk groups

In signal pathway study, differential expression analysis was performed first for gene analysis and specimen with high (*n* = 282) as well as low (*n* = 248) CLRM values were analyzed using limma package R. In gene mutation study, data of genetic changes was downloaded from cBioPortal database. Maftools package of R was used to analyze the quantity as well as quality of mutations in the two CLRM risking parts. Relation analysis among CLRM risking part group and express of PD1, PDL1, CTLA4 and BTLA was conducted. To verify 530 KIRC immunity features of specimens, the express information were imported in CIBERSORT (https://cibersort.stanford.edu/), with an iterative 1000 times in order to estimate the relative ratio of 22 kinds of immunity cells. We then contrasted relative ratios of 22 immunity cells and clinic pathological elements among two CLRM risking parts, with consequences showed presented in the landscape plan. To further clarify immunological and molecule functions among CLRM rising parts, we conducted single sample Gene Set Enrichment Analysis(ssGSEA) on some gene markers and contrasted values among two CLRM risking parts [19–22].

## Results

### Verification of cuproptosis-related lncRNAs in KIRC patients

**Fig. 1:**
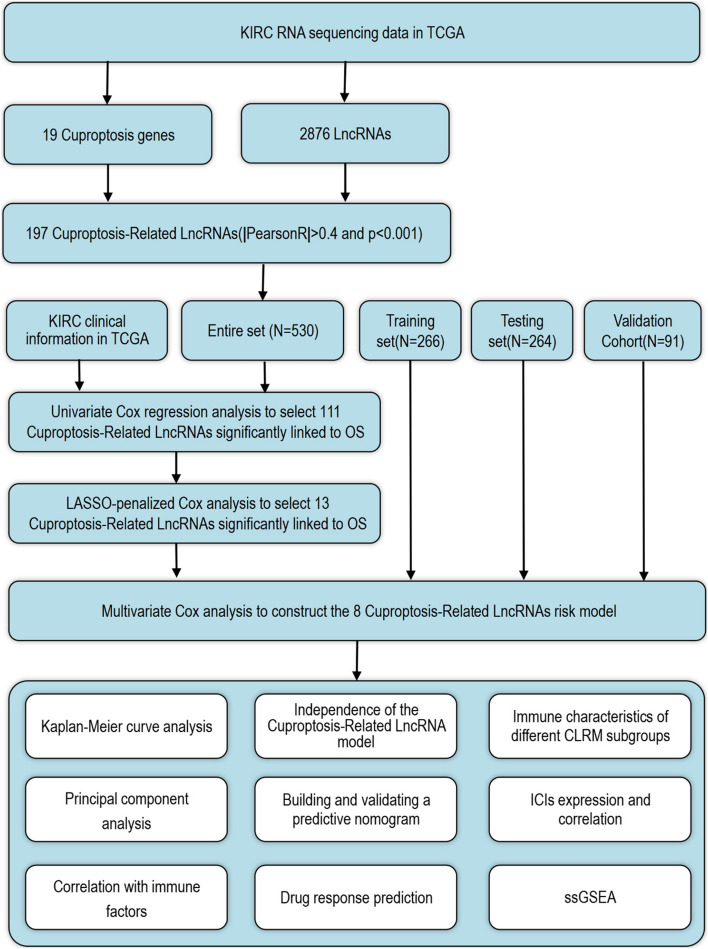
The workflow of the study

**Fig. 2:**
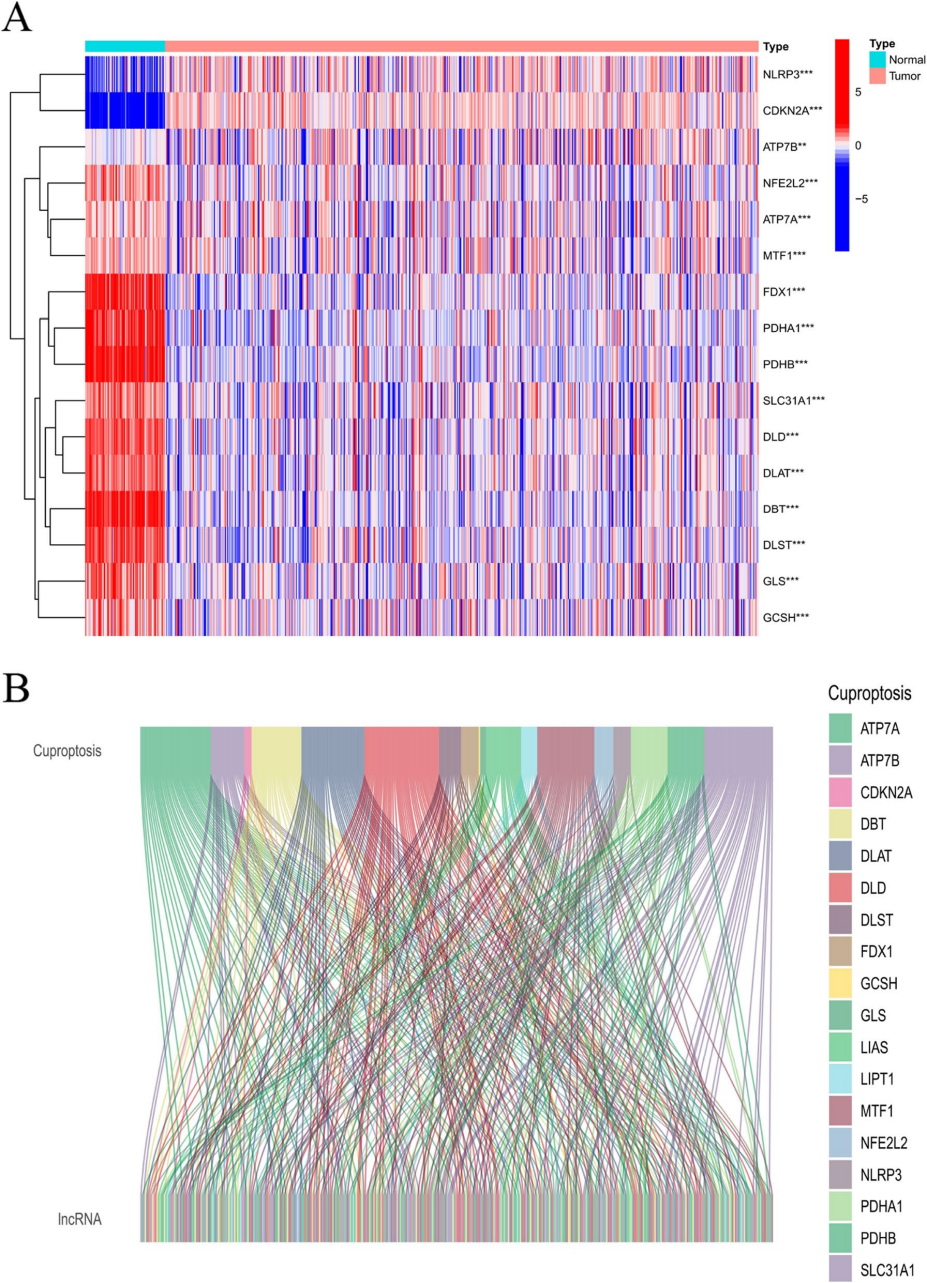
Expressions of 19 cuproptosis-related genes & Identification of lncRNAs in KIRC patients. **A** Heat map (blue: low express standard; red: high express standard) of cuproptosis-related genes among ordinary (N, brilliant green) as well as cancer tissues (T, orange). P sores were indicated as: **P < 0.01; ***P < 0.001. **B** Sankey relation diagram for 19 cuproptosis genes and cuproptosis-related lncRNAs

The specific workflow of risking model establishment and succeeding analysis is indicated in Fig. 1. Matrix expressions of 19 cuprotosis genes as well as 2876 lncRNAs were drew from TCGA database. The RNA standards of the genes are showed as heat maps in Fig. 2A. Cuproptosis-related lncRNAs were defined as one with greater than or identical to 19 Cuproptosis genes remarkably related lncRNA (|Pearson R|> 0.4, *p* < 0.001). Finally, the co-expression network of Cuproptosis-lncRNA was visual by Sankey diagram (Fig. 2B), and 197 lncRNAs were identified as Cuproptosis-related lncRNAs.

### Establishment and validation of a risking model based on cuproptosis-related lncRNAs of KIRC patients

**Fig. 3:**
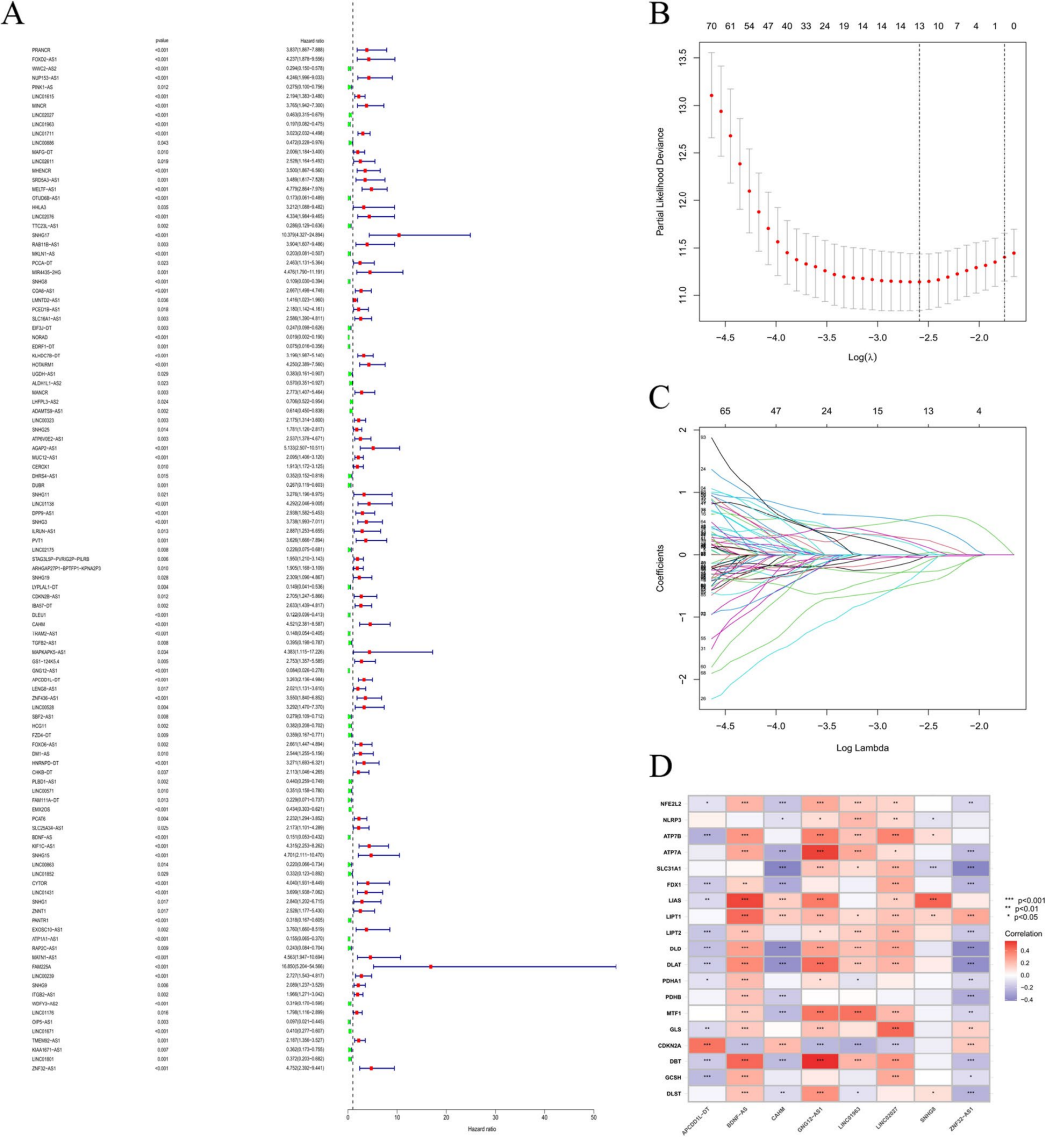
The risking model for KIRC patients according to cuproptosis-related lncRNAs (**A**) Univariate Cox regression study indicated that the selective lncRNAs remarkably related to clinic prognostic. **B** LASSO coefficient curve of 13 OS-related lncRNAs and vertical imaginary line were plotted at the score selected by 10-times cross validation. **C** Regulation parameter (log λ) of OS-related proteins were chosen to cross validate the wrong profile. Based on minimum standard and 1-se standard, vertical imaginary lines were plotted at best score. **D** Heat map for correlation among 19 cuproptosis genes as well as 6 prognosis cuproptosis-related lncRNAs

Univariate Cox regression study was adopted to screen cuproptosis-related prognostic lncRNAs from 2876 cuproptosis-related lncRNAs of KIRC dataset from TCGA database. In the TCGA database, 111 cuproptosis-related lncRNAs were remarkably related to OS (Fig. 3A). LASSO-penalized Cox study is commonly used for various regression study. It can not only enhance prediction precision as well as ability of statistic model, but also make variable choices and simultaneous modulation. The approach is widely used to best selection of features in high dimension information with a poor correlation. The forecast score is prominent and over-fitting is avoided. Therefore, this approach can efficiently identify the most effective predictive markers and generate prognosis indicators that forecast clinic outcomes. The dotted line perpendicular represents the first order score of logarithm L, with the smallest piecewise likelihood deviation. Therefore, 13 cuproptosis-related lncRNAs were chose for succedent multivariate study (Figs. 3B and C). Secondly, multivariate Cox ratio risk regression study was used to differentiate the prognosis proteins. 8 cuproptosis-related lncRNAs (Table S2) were prognosis proteins single related to OS in training part and were applied to build a risking model to evaluate the prognosis risking of patients with KIRC (Table S3). The correlation between cuproptosis genes and cuproptosis-related lncRNAs in entire TCGA set is indicated in Fig. 3D.

**Fig. 4:**
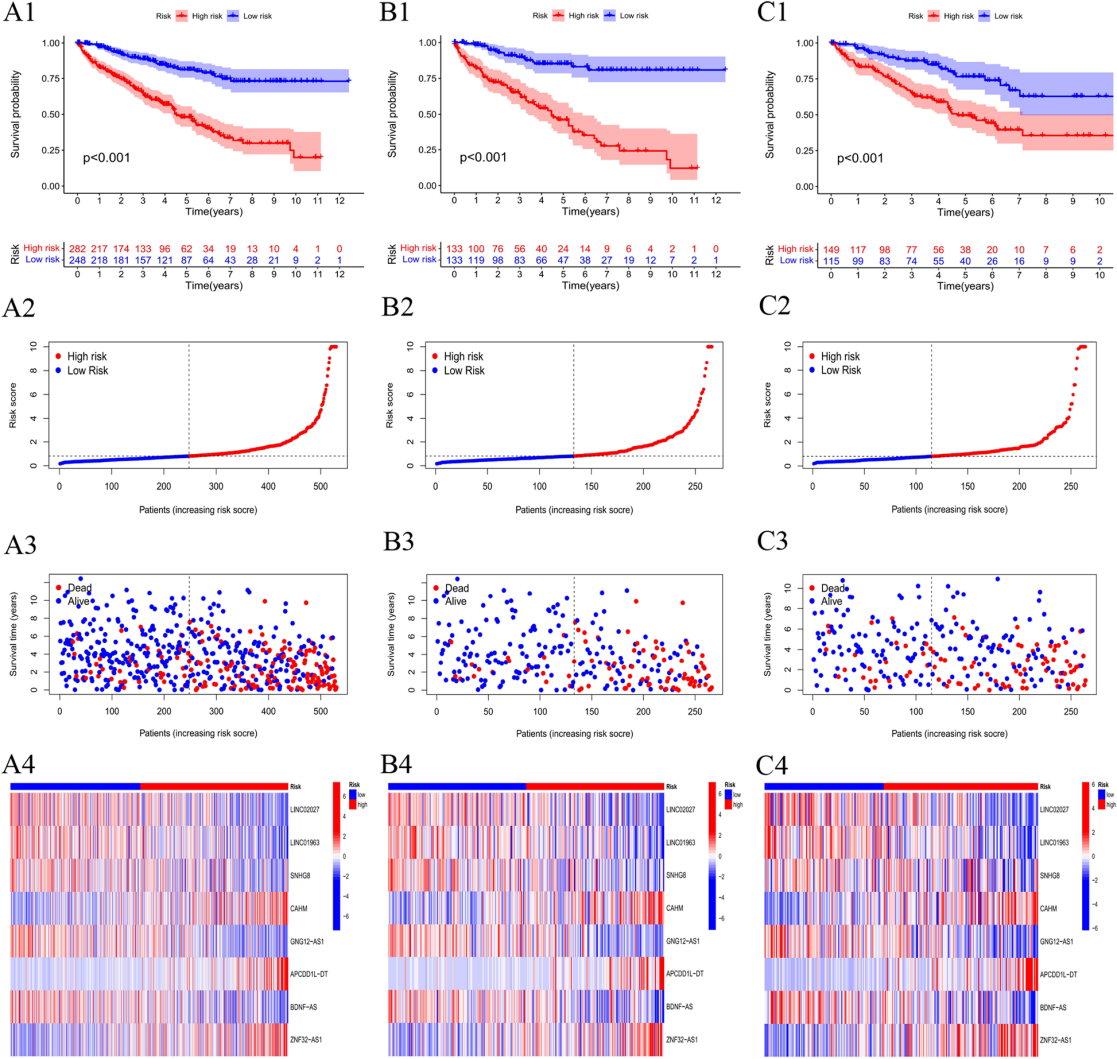
Prognosis score of risking patterns of the CLRM in the TCGA dataset. **A**1 Kaplan–Meier living profiles of OS of patients in high- as well as low-risking parts of entire dataset (**A**2) Distribution of CLRM-based risking value. **A**3 Distinct modes of living condition as well as living time among both groups. **A**4 The express criterions of CLRM for every patient presented by the clustering analysis heatmap. **B**-**C** Relevant results of the training part and testing part

Based on middle of prognostic risking levels, KIRC samples were divided into the low-risk and high-risking parts, which were subjected to Kaplan–Meier survival study. Figures 4A1 present living status of patients in both groups in the whole dataset. The results indicated high risking part had poorer prognostic than low risking part, with a remarkable distinction (*P* < 0.001). Figure 4A2 presents distribution of risk levels of patients in both groups, and Fig. 4A3 presents living condition as well as living time of patients among both groups. The relative express standards of 8 cuproptosis-related lncRNAs for each patient are shown in Fig. 4A4.

In an attempt to detect prognosis ability of the built model, risking values of each patient in training part and the test set were calculated with a uniform formula. Figure 4B-C presents distribution of risking grades, the mode of living condition as well as living time, as well as express of cuproptosis-related lncRNAs in training part (Figs. 4B1–4) as well as test part (Figs. 4C1–4).

**Fig. 5:**
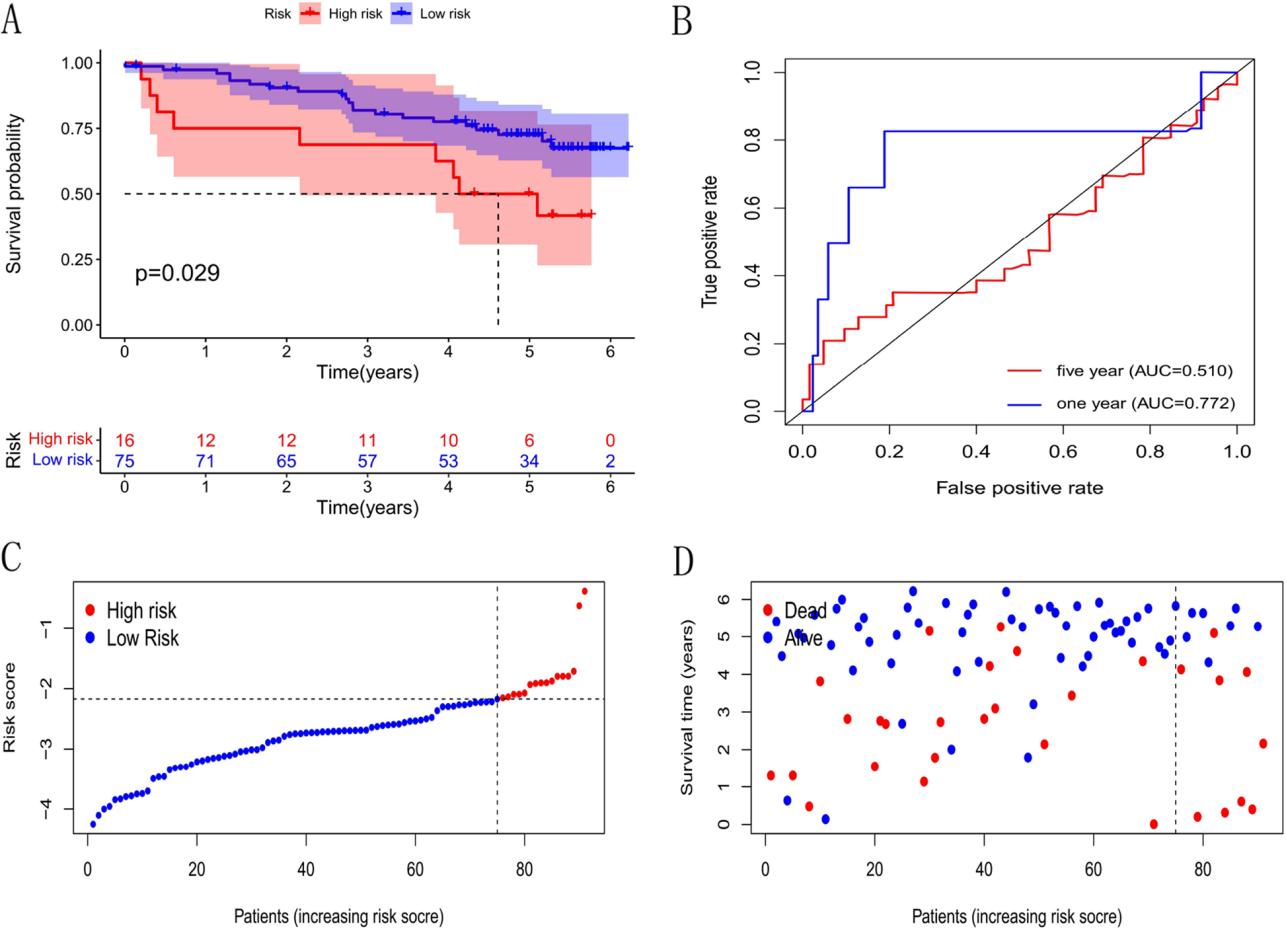
Validation of Independent Cohort. **A** Kaplan–Meier living profiles of OS of patients in high-as well as low-risking parts. **B** ROC curves of clinical features (-1 years, -5 years). **C** Distribution of CLRM-based risking value for Validation part. **D** Different modes of living status as well as living time for Validation set

To further identify precision and practicability of the model, we validated expression profile data of 91 renal cell carcinomas in the ICGC database. The results show that the model still has a good effect on predicting survival time, especially the short-term survival (Fig. 5).

We analyzed OS differences between low and high risking parts, and risking sites across TCGA group, which were layered based on prevailing clinic pathological characters. In terms of age, sexuality, phase as well as grade, OS of the low risking part continued to be better than that of the high risking part (Supplementary Fig. 1).

### Validation of the grouping ability of the CLRM by PCA

**Fig. 6:**
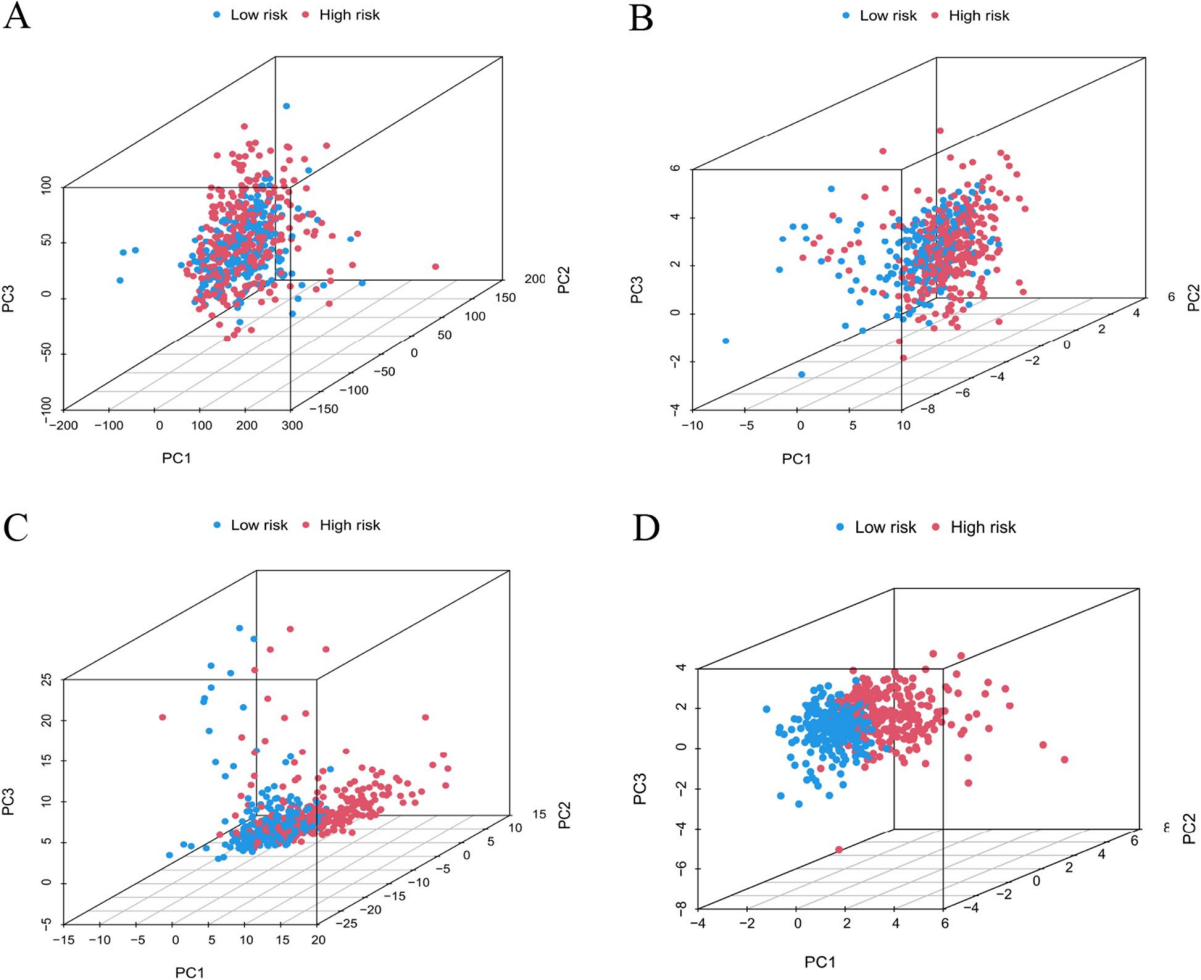
Principal component analysis among both parts according to (**A**) the whole gene expressed curves, **B** 19 cuproptosis genes, **C** 197 cuproptosis-related lncRNAs, and (**D**) the risking model according to the representative curves of CLRM in the whole TCGA part

The PCA of this model was performed to validate distinction among low risking as well as high-risking parts according to total gene expressed curves, 19 cuproptosis genes, cuproptosis-related LncRNAs, and the risking model sorted by expressed curves of CLRM (Figs. 6A–D). As shown in Figs. 6A–C, the distribution of high- as well as low-risking parts is relatively dispersive. But consequences acquired according to this model demonstrated there were differences in the distribution between both groups (Fig. 6D). The consequences suggested that prognosis markers can be distinguished among both groups.

### Evaluation of TMB and tumor immunotherapy response by the CLRM

**Fig. 7:**
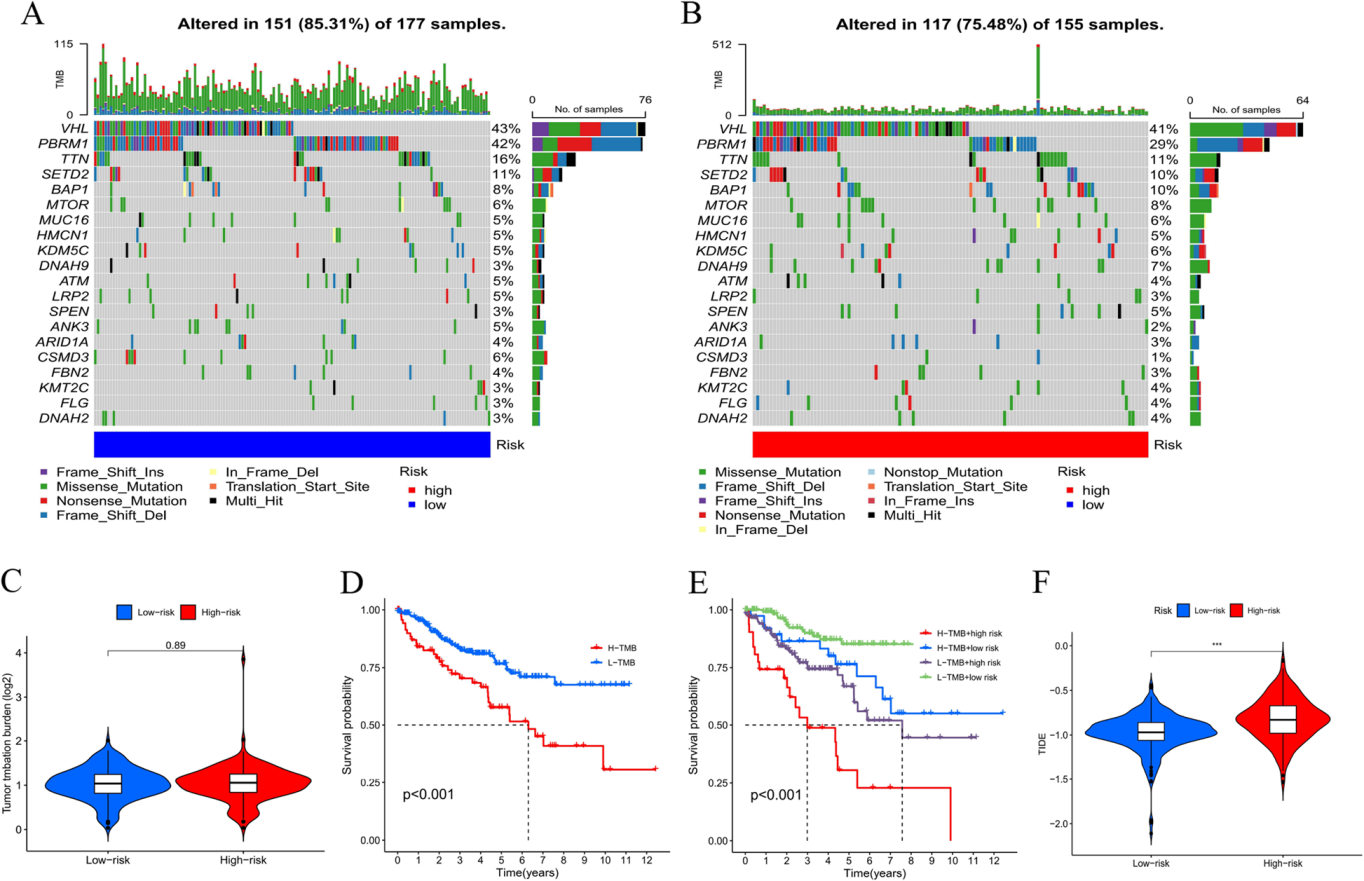
Evaluation of TMB and tumor immunotherapy response by the CLRM. **A**, **B** The mutation data of genes with high mutation frequencies in (**A**) high risking part as well as (**B**) low risking part presented by Waterfall plot. **C** TMB difference in patients of both groups. **D**-**E** Kaplan–Meier profile study of OS of patients sorted based on high/low mutation condition and CLRM. **F** TIDE prediction difference in patients of both groups

The mutation information were studied and summarized by R package maftools. The mutation was stratified according to the predictors of mutation effects. Figures 7A and 7B present the toptwenty driver genes with the highest changing frequency among both subgroups. Then, TMB values were counted based on TGCA somatic mutation information. Results suggested that there was no distinction in cancer mutation burden among low- and high-risking parts (Fig. 7C). Moreover, Kaplan–Meier survival analysis of TMB was conducted in tumor samples. The results in Fig. 7D indicated high-mutation part had a poorer survival prognosis than low-mutation part. As per further analysis, it was found that the high-mutation and high risking part had the worst prognostic, but low-mutation and low risking part had a better prognostic. When the two groups had high-mutation or low-mutation risking, high risking part still had a worse prognostic than the low risking part (Fig. 7E). Subsequently, the relation among CLRM as well as immunity therapy response was explored. Sure enough, it was discovered high risking group was more possible to respond to immunotherapies than the low risking group, which indicated that this CLRM can be used as a sign to predict TIDE (Fig. 7F).These findings were also consistent with our previous results, which suggested that this risk model was effective and stable.

### Immune and molecular characteristics of different CLRM subgroups

**Fig. 8:**
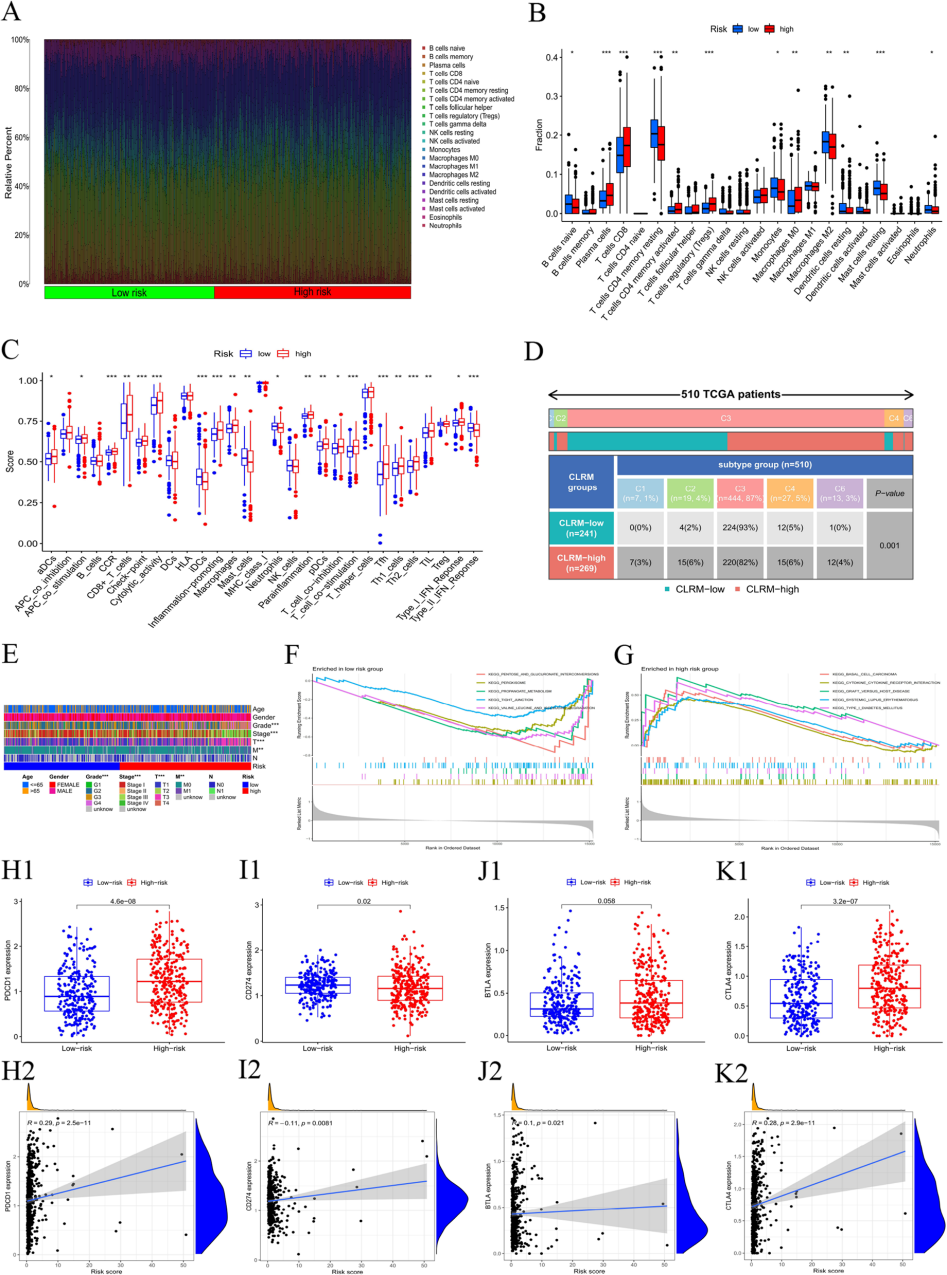
Immune and Molecular characteristics of different CLRM subgroups. **A** Bar plot showed the relative percent of 21 tumor infiltrating immune cells in the high-and low-risking parts. **B**Violin plot showed the difference of the fraction of each immune cells between the two risking parts. **C**-**D** Immune function and Immune Characteristics related to the immune landscape. I Differences in clinical characteristics between high and low risking parts. **F** and **G** Gene set enrichment analysis based on Kyoto Encyclopedia of Genes and Genomes (KEGG) of low-risk group (**F**) and high-risk group (**G**). **H–K** The differences between the two groups in the expression and the correlation of the common immune checkpoints

To analyze the composition of immune cells in different CLRM subgroups, we used the Wilcoxon test to compare the distribution of immune cells in different CLRM subgroups. We found that Plasma cells, T cells CD8, T cells CD4 memory activated, T cells regulatory (Tregs) and Macrophages M0 were more abundant in the CLRM-high risk group, while B cells naïve,Plasma cells,T cells CD4 memory resting,Monocytes, Macrophages M2, Dendritic cells resting,Mast cells resting and Neutrophils were more abundant in the CLRM-low risk group (Fig. 8A-B). Characteristics related to the immune landscape are displayed in Fig. 8C-D.

Analysis of differences in clinical features between high and low risking parts (Fig. 8E) showed significant differences between clinical stage, grade, T and M. GSEA was performed to determine the gene sets enriched in different CLRM subgroups. The gene sets of the CLRM-low samples were enriched in Pentose and Glucuronate interconversions, Peroxisome, Propanoate Metabolism, Tight Junction and Valine Leucine and Isoleucine Degradation (Fig. 8F), while the gene sets of the CLRM-high samples were enriched in Basal Cell Carcinoma, Cytokine Receptor Interaction, Graft Versus Host Disease, Systemic Lupus Erythematosus and Type I Diabetes Mellitus pathways (Fig. 8G) (*p* < 0.05, FDR < 0.25). To further explore the difference in the response to immunotherapy between the two groups, we compared the differences in the expression and the correlation of immune checkpoints. As shown in Fig. 8H-K, programmed cell death (PD-1) and B- and T-lymphocyte attenuator (BTLA) were elevated in the high-risk group, while PD ligand 1 (PD-L1, CD274) was lowered in the low-risk group.

### Evaluation of the prognostic risk model and clinical features of KIRC

**Fig. 9:**
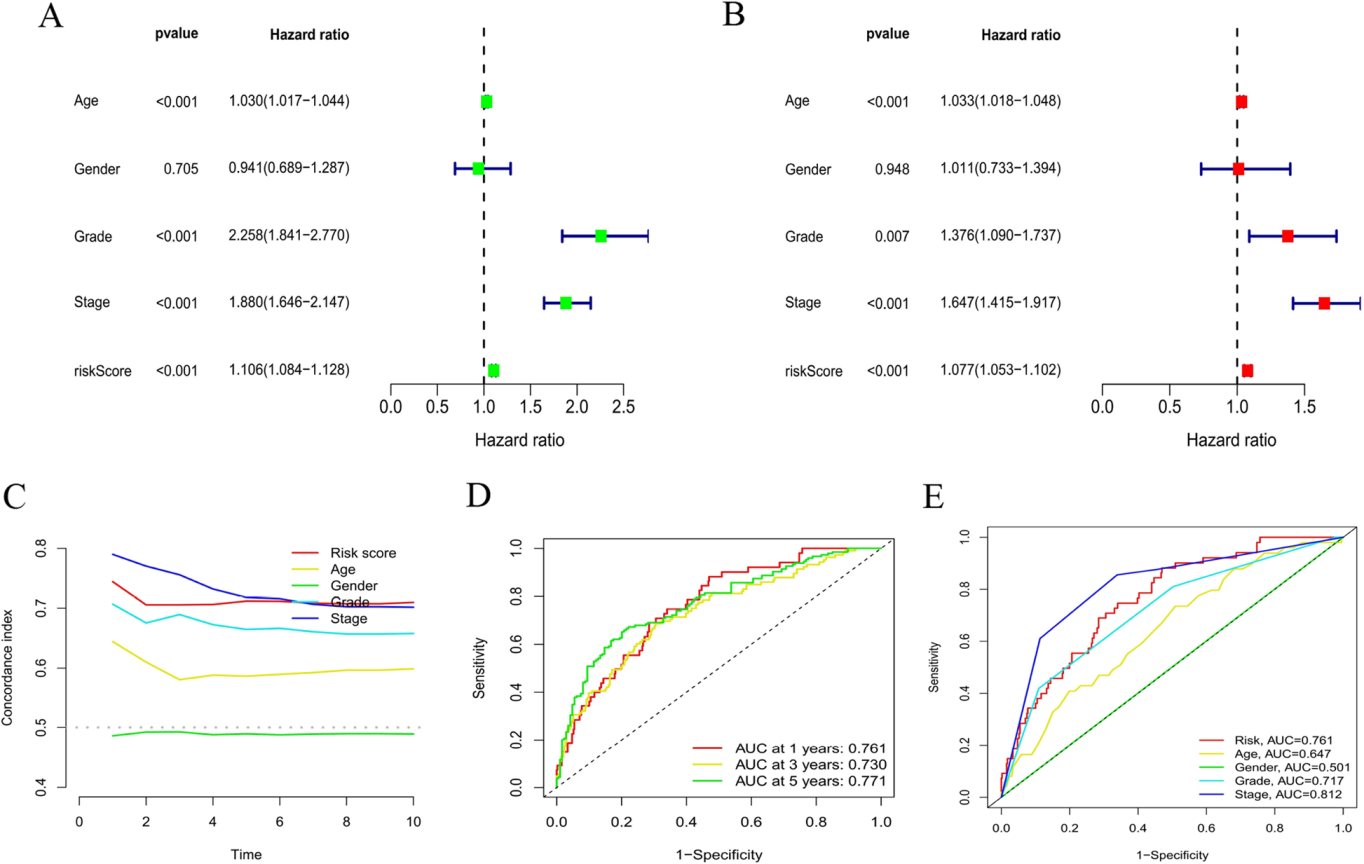
Assessment of the prognostic risk model and clinical features of KIRC in the entire TCGA set. **A-B** Univariate and Multivariate analyses of the clinical features and risk scores with the OI(C) Concordance indexes of the risk scores and clinical features. (D-E) ROC curves of the clinical features and risk scores

In this study, univariate and multivariate Cox regression analyses were conducted to evaluate whether the risk model had independent prognostic characteristics of KIRC. The univariate COX regression analysis results showed that the odds ratios of Hazard Ratio(HR) and 95% CI were 1.106 and 1.084–1.128 (*P* < 0.001) (Fig. 9A). The multivariate Cox regression analysis results showed that HR was 1.077 and 95% CI was 1.053–1.102 (*P* < 0.001), respectively (Fig. 9B). It suggested that the risk model can effectively predict the prognosis independent of other clinical features. The concordance index and the area under ROC curve (AUC) of risk score were assessed to properly evaluate the uniqueness and sensitivity of risk score in predicting the prognosis of KIRC patients (Figs. 9D-E). With the extension of time, the concordance index of risk score gradually increased with the risk level and became higher than that of other clinical factors. It suggested that the risk level of this model was effective in predicting the prognosis of KIRC patients (Fig. 9C). The AUC of the risk level also became higher than that of most other clinicopathological factors. It suggested that CLRM can be reliably applied in the prognostic risk model for KIRC patients.

### Construction and evaluation of the prognostic nomogram

**Fig. 10:**
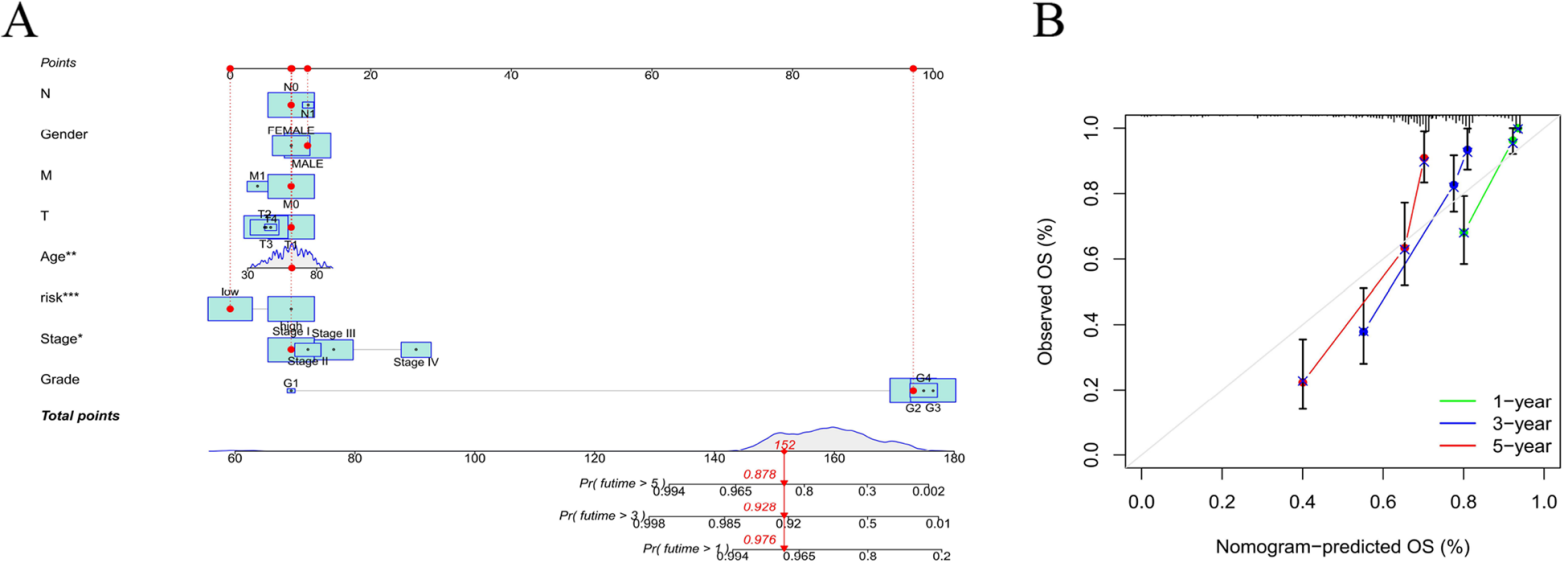
Construction and evaluation of a prognostic nomogram. **A** The likelihood of the 1-, 2-, and 3-year OS predicted by the nomogram. **B** The likelihood of the 1-, 2-, and 3-year OS predicted the calibration plot of the nomogram

A nomogram incorporating the risk levels and clinical risk features was constructed to predict the OS of patients at 1, 2 and 3 years. According to the nomogram, the risk level of the prediction model showed a significant predictive ability through a comparison with clinical factors (Fig. 10A). Relevant diagrams showed that there was favorable concordance in the observation and prediction rates of OS at 1, 2 and 3 years (Fig. 10B).

### Identification of potential drugs for CLRM

In order to identify potential drugs for CLRM in the treatment of KIRC patients, the pRophetic algorithm was adopted to estimate the treatment response based on the half maximal inhibitory concentration (IC50) of each sample provided in GDSC. A total of 102 compounds were identified and there were significant differences in estimated IC50 between both groups. Supplementary Fig. 2 presents partial sensitive compounds.

## Discussion

Renal cell carcinoma (RCC) is a malignancy from renal tubular epithelium, and its incidence ranks third among all tumors in the urinary system, with an upward trend with each passing year [23]. Although surgical resection is the most effective method in the treatment of RCC, the majority of patients have progressed to the middle and advanced stages at the moment of diagnosis. Besides, such tumors are not sensitive to radiotherapies, chemotherapies and immunotherapies, and short-term drug resistance may occur during the application of targeted therapies. Thus, RCC patients usually have a poor prognosis [24, 25].

Therefore, the prediction and treatment of RCC can be promoted by exploring the critical genes and molecules that affect the occurrence and development of KIRC and constructing a stable prognosis model. Recently, the identification of a new cell death mechanism, namely cuproptosis [11], provides an important approach to inducing cell death, which is different from such traditional cell death methods as apoptosis, ferroptosis, pyroptosis and necroptosis. There are double roles for copper. Specifically, copper fulfills an essential function as a cofactor of enzymes for all animals; however, even a moderate intracellular concentration of copper may be toxic and even induce cell death [26]. As is revealed from existing studies, cuproptosis is mediated by an ancient mech–nism—protein lipidation. Lipidated proteins are mainly distributed in the tricarboxylic acid(TCA) cycle, in which lipidation may be required for the function of enzymes [27, 28]. Besides, the relationship between mitochondrial metabolism and cuproptosis sensitivity is further explained in some studies. Specifically, the cells with active respiration and TCA cycles increase the lev17apidatedidated TCA enzymes (especially PDH complex); sulfonyl directly binds copper, which results in the aggregati17apidatedidated proteins and the loss of proteins containing Fe-S clusters, thus inducing heatshockprotein70(HSP70) reflecting acute proteotoxic stress [11]. However, the growth and proliferation of tumor cells are closely related to the TCA cycle and other basal metabolic processes [29, 30]. Based on that, it can be boldly speculated that cuproptosis may play a certain role in RCC.

After the KIRC expression profile data, clinical data and mutation spectrum data are downloaded from the TCGA database to conduct integrated analysis, the differential expression of 19 cuproptosis-related genes from normal kidney tissues and tumor tissues is explored at first. Surprisingly, it can be found that there are 16 differentially expressed genes (DEGs) among these 19 cuproptosis-related genes (Fig. 2A), including 14 genes that are highly expressed in normal samples, accounting for more than 90% of DEGs. It suggests that cuproptosis is a low-level activity in RCC cells. If cuproptosis is activated, will the growth of KIRC cells be inhibited? This shall be a question worthy of further exploration!

As per the systematic analysis of lncRNA expression profile, there are many abnormally expressed lncRNAs in RCC [31, 32], which could cause changes in protein expression and function and corresponding cell signaling pathways. Additionally, these abnormally expressed lncRNAs closely correlate with the occurrence, development, diagnosis, prognosis and drug resistance of RCC [33, 34],

NLRP3 is a member of the cuproptosis gene family. Related studies found that LincRNA-CoX2, previously known as a mediator of activation and suppression of immune gene expression in innate immune cells, binds TO NF-κB P65 and promotes its nuclear translocation and transcription, regulating the expression of inflammasome sensor NLRP3 and adaptor ASC [35]. Kumar A et al. found that the expression of NLRP3 and its downstream components (caspase-1 and IL-1β) were enhanced in ccRCC, and LSD2 may be involved in the regulation of NLRP3 immunosomes in cancer cells, which could be a potential target for the treatment of ccRCC [36].

Another related study showed that pyruvate dehydrogenase E1β subunit (PDHB) may be involved in the occurrence and development of colorectal cancer (CRC) under the regulation of LncRNA maternally expressed gene 3 (MEG3) [37]. Although there are not many reports on cuproptosis gene-related long non-coding RNA at present, long non-coding RNA, as an important epigenetic regulator, is likely to play an important role in the expression of cuproptosis-related genes [38].

In this study, an independent prognostic model based on cuproptosis-related lncRNAs is constructed according to the role of cuproptosis and lncRNAs in KIRC. Further, the potential effective drugs for treating KIRC are also investigated based on this model. A total of 197 cuproptosis-related lncRNAs are identified from the TCGA database, which can be employed to explore the prognostic function of cuproptosis-related lncRNAs. As per the results from the TCGA database, the prognostic value of 13 cuproptosis-related lncRNAs is validated, among which 8 cuproptosis-related lncRNAs can be employed to construct the cuproptosis-related lncRNA model to predict the OS of KIRC patients. Moreover, KIRC patients are divided into the high-risk group and the low-risk group based on the median of prognostic risk scores. The results indicate that the high-risk group has a worse prognosis. As per the multivariate Cox regression analysis results, the cuproptosis-related lncRNA model is an autologous risk factor for OS. The ROC analysis results suggest that this model is more effective than most conventional clinical features in predicting the OS of KIRC patients. Furthermore, a nomogram is also plotted to present the perfect concordance between the observation and the prediction 1-, 3-, and 5-year OS rates of the operating system. Finally, there is excellent concordance in the prediction 1-, 3-, and 5-year OS rates of the operating system. There is a higher accuracy for the risk model based on 8 cuproptosis-related lncRNAs independently related to OS of KIRC patients has a higher accuracy. Hence, this prediction model can be employed to identify new biomarkers for subsequent studies.

Additionally, the TIDE algorithm is adopted to predict the likelihood of the immunotherapeutic response. The results indicate that the high-risk group has a larger immune response rate than the low-risk group, which also suggests that immune-related drugs may have better efficacy in the high-risk group in the prediction model. This finding also provides guidance values for the application of immune-related drugs.

Furthermore, the immune and molecular characteristics of different subgroups are also analyzed under the model. The results suggest that there are certain differences in the enrichment and infiltration of immune cells between the high-risk and low-risk groups. Meanwhile, the expression and correlation of common immune-related genes such as PD1 and PD-L1 are also analyzed. The results indicate that there are significant differences in the expression of these important immune-related genes between the high-risk and low-risk groups. In addition, the expression of these genes positively correlates with risk scores.

This model has been verified through the datasets of the TCGA database. However, the ICGC RCC dataset are combined as an external cohort to verify the accuracy and practicability of this model. The survival analysis results demonstrate that there is a significant difference between the high-risk and low-risk groups. Therefore, this model can effectively predict the survival prognosis of KIRC patients.

As is known to all, pathological stage and grade are the decisive factors for the prognosis of KIRC patients. However, the same clinical stage and grade of tumors are not equal to the same prognosis. Therefore, it is of great significance to explore more comprehensive and specific predictive indicators or biomarkers. This cuproptosis-related lncRNA model has been constructed to provide a novel method for predicting the prognosis of KIRC patients. These findings also provide a new insight for exploring the modification process and mechanism of cuproptosis in lncRNAs. In this study, multiple methods are adopted to verify this new model, and hence the optimal model can be properly selected and applied.

However, there are still some limitations in this study. For instance, the biological mechanism of cuproptosis-related lncRNAs is not fully clarified in this model. Therefore, it is necessary to explore the role of lncRNAs and their interaction with cuproptosis-related genes. In summary, the findings in this study provide novel insights for predicting the survival and prognosis of KIRC patients, which may contribute to revealing the process and mechanism of cuproptosis-related lncRNAs. Furthermore, some potentially effective drugs are also preliminarily identified after the construction of this immunotherapy-sensitive model, which brings some implications for the treatment of KIRC patients.

## Supplementary Information

**Additional file 1: Supplementary Figure 1. **Kaplan-Meiercurves of OS differences stratified by age, gender, tumor grade and stagebetween both groups in the entire TCGA set. 

**Additional file 2: Supplementary Figure 2. **Partial sensitive compounds.

**Additional file 3: Table S1.** Thegene names of the 19 cuproptosis-related genes.

**Additional file 4: Table S2. **The names of the 8 cuproptosis-related lncRNAsin the model.

**Additional file 5: Table S3. **Theclinical features between the entire set, training set and the testing set inthe risk model.

## Data Availability

The datasets generated and/or analysed during the current study are available in the [TCGA] repository, [https://portal.gdc.cancer.gov/repository] & [ICGC] repository, [https://dcc.icgc.org/repositories].
