# Supplementary figures and images for: Exploration of the role of Cuproptosis genes and their related long non-coding RNA in clear cell renal cell carcinoma: a comprehensive bioinformatics study
Source: BMC Cancer. 2022 Nov 6;22:1141. doi: 10.1186/s12885-022-10278-z (PMC9637316; doi:10.1186/s12885-022-10278-z)

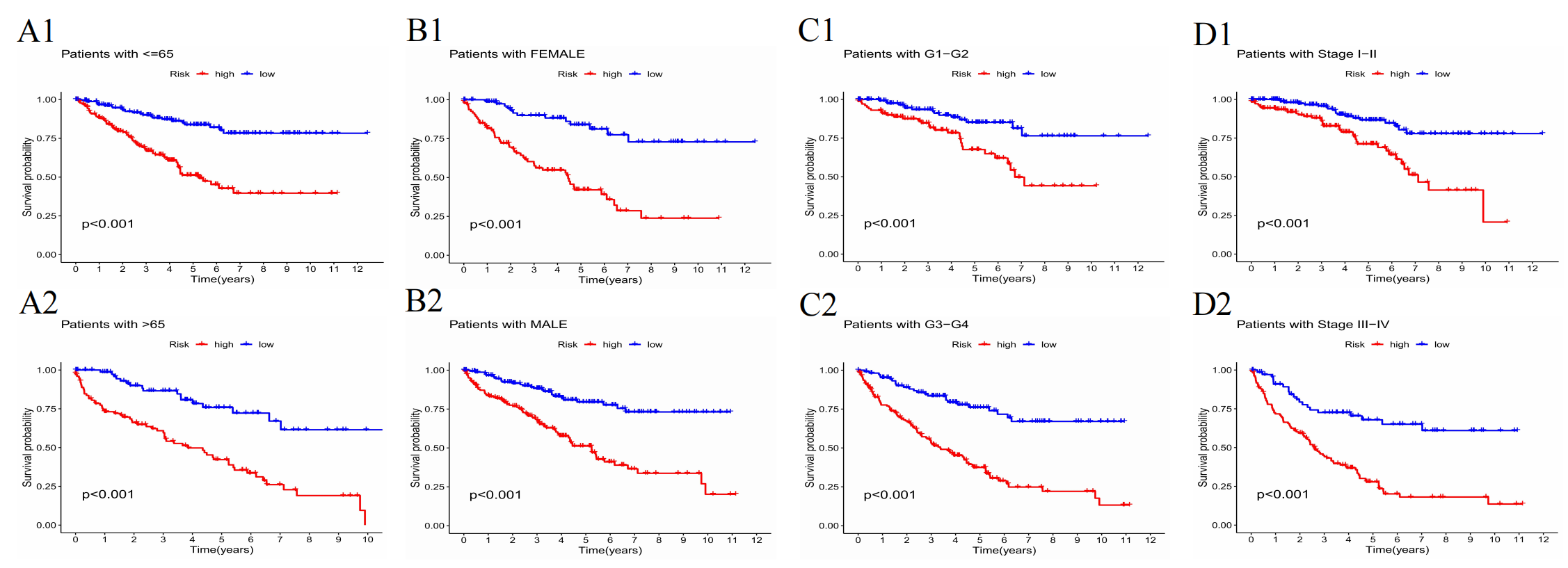

Supplement: Supplementary file 1 — Additional file 1: Supplementary Figure 1. Kaplan-Meiercurves of OS differences stratified by age, gender, tumor grade and stagebetween both groups in the entire TCGA set. [file 12885_2022_10278_MOESM1_ESM.tif]

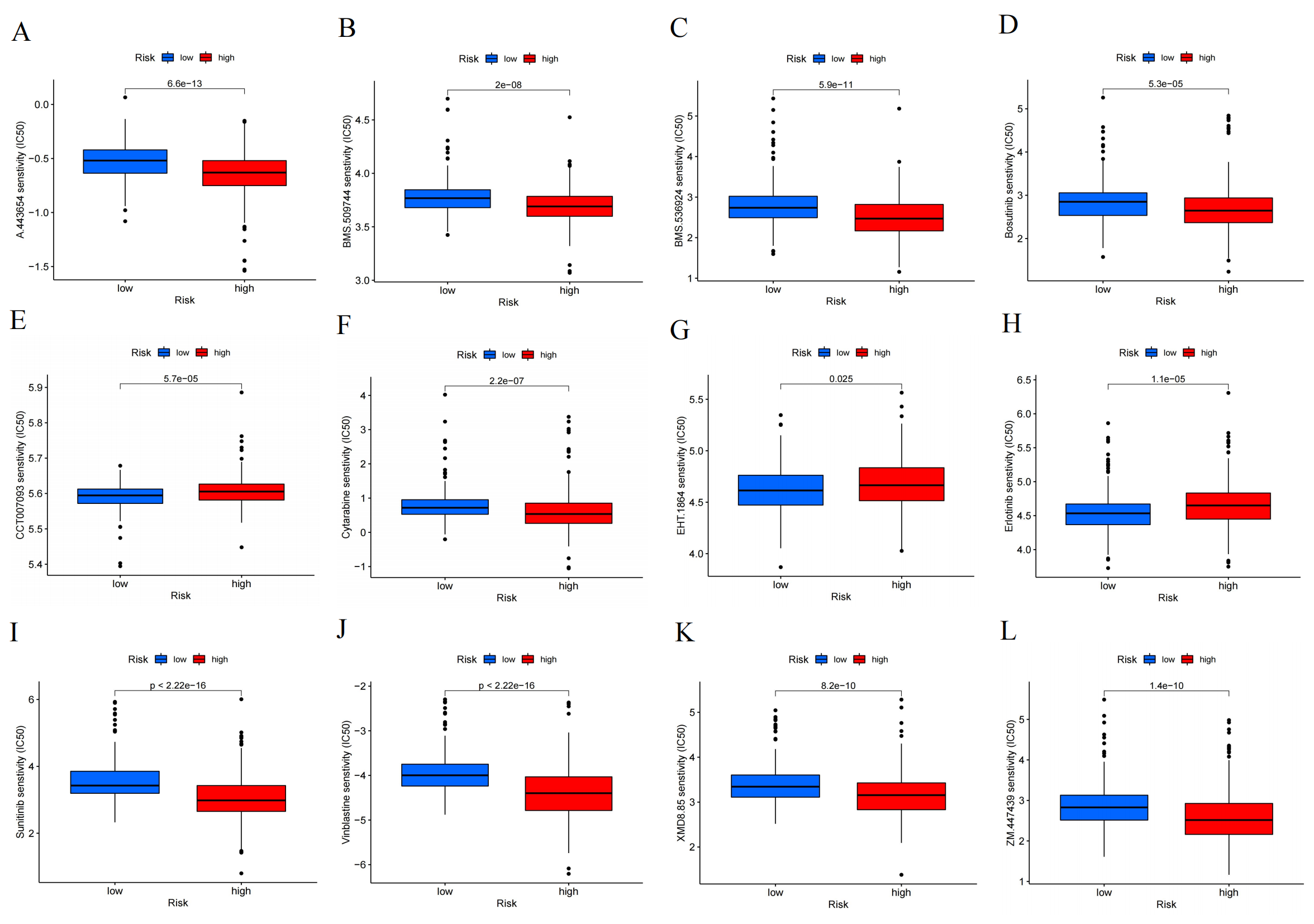

Supplement: Supplementary file 2 — Additional file 2: Supplementary Figure 2. Partial sensitive compounds. [file 12885_2022_10278_MOESM2_ESM.tif]
